# Botanical from *Piper capense* Fruit Can Help to Combat the Melanoma as Demonstrated by *In Vitro* and *In Vivo* Studies

**DOI:** 10.1155/2021/8810368

**Published:** 2021-01-18

**Authors:** Brice E. N. Wamba, Paramita Ghosh, Armelle T. Mbaveng, Sayantan Bhattacharya, Mitra Debarpan, Saha Depanwita, Mustafi Mitra Saunak, Victor Kuete, Nabendu Murmu

**Affiliations:** ^1^Department of Signal Transduction and Biogenic Amines, Chittaranjan National Cancer Institute, 37, S.P. Mukherjee Road, Kolkata 700026, India; ^2^Department of Biochemistry, Faculty of Science, University of Dschang, Dschang, Cameroon; ^3^Department of Pathology, Chittaranjan National Cancer Institute, 37, S. P. Mukherjee Road, Kolkata 700026, India

## Abstract

*Piper capense* belongs to Piperaceae family and has long been used as a traditional medicine to treat various diseases in several parts of Africa. The present study aims to investigate the effect of *Piper capense* fruit extract (PCFE) alone and in combination with dacarbazine on metastatic melanoma cell line B16-F10 and *in vivo* in C57BL/6J mice. Cytotoxic effects of PCFE alone and in association with dacarbazine on B16-F10 cells were studied by 3-(4, 5-dimethylthiazol-2-yl)-2, 5-diphenyl tetrazolium bromide (MTT) assay and colony formation assay. Wound healing assay, immunofluorescence staining, and western blot analysis were performed to evaluate the individual and combined effect of PCFE and dacarbazine on epithelial-mesenchymal transition (EMT). For *in vivo* studies, C57BL/6J mice were subcutaneously injected with B16-F10 cells (5 × 10^5^ cells/mL), and the effect of PCFE and dacarbazine was studied on tumor development. The alteration of EMT was evaluated by targeting E-cadherin, vimentin, and CD133 in PCFE alone and in combination with dacarbazine-treated tumor tissues by western blot analysis. Phytochemical screening of PCFE reveals the presence of certain secondary metabolites. Our results showed that PCFE alone and in association with dacarbazine has a good activity in preventing B16-F10 melanoma cell progression and clonogenicity. This extract also regulated EMT. *In vivo* results showed that PCFE (100 mg/kg body weight) reduced tumor size in C57BL/6J mice along with the decrease in the expression of vasculogenic mimicry (VM) tubes as well as an improvement in the qualitative and quantitative expression of markers involved in EMT. Our study suggests that PCFE may be useful for managing the growth and metastasis of melanoma.

## 1. Introduction

Cancer is increasingly recognized as a critical public health problem worldwide, specifically in some parts of Africa where people are poor and do not have the financial means to obtain adequate treatment even though the survival rates are lower compared to other countries [1].

The latest World Health Organization (WHO) global data show 18.1 million new cases and 9.6 million cancer deaths in 2018 [2]. It is recognized as the leading and the second leading cause of death, respectively, in economically developed and developing countries [3]. WHO Bulletin in 2018 reports 15769 cases of cancer detected against 10533 cases of deaths or more than half of the incidence in Cameroon [2]. There are more than a hundred different cancers depending on the organ affected. Malignant melanoma is the most aggressive form of skin cancer. About 96480 new cases of melanoma have been diagnosed (57220 in men and 39260 in women) and 7230 cases of death (4740 in men and 2490 in women) from this disease in the United States in 2019 according to the work of Siegel et al. [4]. Melanomas which are not of epithelial origin develop from neural crest-derived pigmented melanocytes [5]. In Africa and more particularly in Cameroon, its incidence in 2018 has been estimated at 148 cases against 89 cases of death [2].

These growing burdens in developing countries are caused by not only etiological factors [6] but also risk factors such as genetics, population growth and ageing, urbanisation, and the adoption of new lifestyles (smoking, alcoholism, and lack of physical exercise). This has led to a rapid increase in incidence, environmental pollution, lack of preventive measures, delay in diagnosis, and a deficit of health workers trained in oncology. If adequate measures are not taken quickly, cancer mortality will continue to increase at the same rate as incidence [7, 8]. The scientific community, in its quest to find ways and means to reduce the morbidity and mortality rate associated with this disease, has set up several treatment strategies. These strategies include chemotherapy, radiotherapy, and surgery which are the most important and have given rise to the hope of eradicating this disease, though it was later on discovered that cancer cells were capable of developing resistant mechanisms to overcome the lethal action of conventional drugs (chemotherapy) given that it is the most widely used treatment method.

Cancer can be subdivided into several types according to the causal agent and the affected organ. Melanoma is particularly common among Caucasians, especially Northern and North-Western Europeans, living in sunny climates. There are higher rates in Oceania, North America, Europe, Southern Africa, and Latin America. This geographic pattern reflects the primary cause, ultraviolet light (UV) exposure in conjunction with the amount of skin pigmentation in the population. A crucial conundrum that goes hand in hand with tumor aggressiveness and poor prognosis of patients is drug resistance. Many tumors, especially melanoma, have the tendency to show resistance against various chemotherapeutic drugs that make the treatment difficult at best [9]. Although the general mode of action for drug resistance is thought to be achieved by the ATP-binding cassette (ABC) transporter system [10] present in the cancer stem cell (CSC) population, the molecular signalling is different in melanoma. Dacarbazine, a potent alkylating agent, is considered the gold standard for melanoma treatment. But the response rate of the drug is only 15–20% [11], and the possible mechanism of evasion is mediated by the upregulation of interleukin-8 (IL-8) and vascular endothelial growth factor (VEGF) expressions, two major proteins that regulate angiogenesis, drug resistance, and tumor cell growth by an autocrine mechanism [12–14].

Besides being the most widely used chemotherapeutic agent for eliminating malignant melanoma, the response rate and duration of dacarbazine treatment are disappointing due to the resistant property of the cells and it is still uncertain whether combination therapies are superior to the single-agent dacarbazine [15, 16] in various randomized phase III studies. In 2018 in Africa, melanoma of skin cancer caused 6629 new cases and 4143 deaths [2].

Cancer cells have developed resistance to existing conventional drugs over time, but also signs of toxicity have been observed; for example, doxorubicin, a widely used chemodrug, causes renal and cardiac toxicity [17–20], and 5-fluorouracil, a common chemotherapeutic agent, is known to cause myelotoxicity and cardiotoxicity [21, 22]. All of these justify the urgency in the search for naturally occurring anticancer drugs with fewer side effects and designed to overcome the resistance problem. Several studies have already been carried out with plant extracts in relation to melanoma cell line B16-F10; for example, Pandey [23], showed the *in vivo* antitumor potential of extracts from the different parts of *Bauhinia variegata* Linn. Rajasekar et al. [24] showed the anticancerous effect of *Lithospermum erythrorhizon* extract *in vitro* and *in vivo*. Uscanga-Palomeque et al. [25] showed the inhibitory effect of *Cuphea aequipetala* extracts on melanomas *in vitro* and *in vivo*. In coherence with these findings, a keen interest in a Cameroonian pharmacopoeia plant called *Piper capense* from the Piperaceae family was taken into consideration due to its therapeutic virtues, most especially in the treatment of several illnesses like cancer when used in the form of formulation [26, 27]. *Piper capense* L. is traditionally used in Cameroon to treat cancers [28]; the aerial part of *Piper capense* L. is traditionally used in the Comoro Islands for diarrhoea and cough [29], with their traditional use in South Africa for the treatment of wounds, vaginal discharge, infertility, sore throat, and tongue sores. Both the aerial part and the plant roots when boiled are also used against malaria in Kenya [30]. Nevertheless, its *in vitro* cytotoxic activity on several types of cancer cell lines, notably methanolic extract against CCRF-CEM leukemia cell lines (inhibitory concentration 50% (IC_50_): 6.95 *µ*g/mL), HL60 (IC_50_: 8.16 *µ*g/mL), HL60AR (IC_50_: 11.22 *µ*g/mL), and CEM/ADR5000 (IC_50_: 6.56 *µ*g/mL), breast adenocarcinoma cell lines MDA-MB231 (IC_50_: 4.17 *µ*g/mL) and MDA-MB231/BCRP (IC_50_: 19.45 *µ*g/mL), colon carcinoma cell lines HCT116 p53+/+ (IC_50_: 4.64 *µ*g/mL) and HCT116 p53−/− (IC_50_: 4.62 *µ*g/mL), glioblastoma cell lines U87MG (IC_50_: 13.48 *µ*g/mL) and U87MG.ΔEGFR (IC_50_: 7.44 *µ*g/mL), has been demonstrated [28, 31]. The activities of *P. capense* MeOH and aqueous extracts against *Mycobacterium tuberculosis* and *C. albicans* with minimal inhibitory concentrations (MICs) of 512 *μ*g/mL and 0.56 *μ*g/mL, respectively [32, 33], have been demonstrated.

Despite all these studies and the results found in the literature, no report on *Piper capense* extract on melanoma cell lines has been documented *in vitro* as well as *in vivo*. Hence, this work aims to evaluate the *in vitro* and *in vivo* anticancer effects of the methanol extract of PCFE alone and in combination with dacarbazine on B16-F10 murine melanoma.

## 2. Material and Methods

### 2.1. Collection and Identification of Plant Material

The fruits of *Piper capense* Linn (Piperaceae) were purchased from the Dschang City main market, in the Menoua Division of the West Region of Cameroon. The plant was subsequently identified and authenticated at the National Herbarium of Cameroon (NHC) by Mr. Fulbert Tadjouteu, Yaoundé, where a sample was deposited and registered under reference number 6018/SRF-Cam.

### 2.2. Preparation of the PCFE

The plant was cleaned and ground and the powder obtained was macerated in methanol in the proportions 1 : 3 (m/v) for 48 hours at room temperature followed by filtration using Whatman No.1 paper. The filtrate obtained was concentrated using a rotary evaporator under reduced pressure (BÜCHI R-200) at 40°C where the crude extract was obtained. The extract was thereafter lyophilized and stored at −20°C for future use.

### 2.3. Chemicals, Antibodies, and Cell Line

3-(4,5-Dimethylthiazol-2-yl)-2, 5-diphenyl tetrazolium bromide (MTT) (Merck) has been used for the revelation of viable cells, Dimethylsulfoxide (DMSO) (Merck) was used to dissolve the formazan crystals formed, Dulbecco's modified Eagle medium (DMEM) and high glucose culture medium supplemented with fetal bovine serum (FBS) (Gibco) were used for the cultivation and maintenance of the B16-F10 cell line, and penicillin/streptomycin (Invitrogen) was used for the preparation of culture media; 4′, 6-diamino-2-phenylindole (DAPI) (LB097-10MG) was purchased from HIMEDIA Laboratories (Mumbai, India) and used as a nuclear counterstain; dacarbazine was purchased from Celon Laboratories (Telangana State, India) and used as reference chemotherapeutic drug.

The aqueous solution of dacarbazine was freshly prepared and exposed to sunlight at least one hour before any experiment to activate the chemotherapeutic drug.

Antibodies vimentin (mouse IgG1; NBP2-32910) and CD133 (rabbit IgG; NB120-16518) were purchased from Novus Biologicals (10730 E. Briarwood Avenue Centennial, CO 80112); E-cadherin (rabbit IgG; sc-7870), *β*-actin (rabbit IgG; sc-47778), and CD31 (mouse IgG) (sc-1506) were purchased from Santa Cruz Biotechnology (Bergheimer Str. 89–2, 69115 Heidelberg, Germany, Europe). All secondary antibodies (anti-mouse IgG-HRP: sc-2031, anti-rabbit IgG-HRP: sc-2004, and anti-mouse IgGk BP-PE: sc-516141) were purchased from Santa Cruz Biotechnology (Bergheimer Str. 89–2, 69115 Heidelberg, Germany, Europe). For periodic acid-Schiff staining (PAS), periodic acid was purchased from Merck (Massachusetts, USA); Schiff's reagent and hematoxylin were purchased from SRL (India).

### 2.4. Cell Culture

The cell line B16-F10 murine melanoma was obtained from the National Centre for Cell Science (NCCS), Pune, India. It was cultured and maintained in Dulbecco's modified Eagle medium (DMEM) and a high glucose culture medium supplemented with 10% fetal bovine serum (FBS) and 1% penicillin/streptomycin in an incubator at 37°C in an atmosphere containing 5% CO_2_. All experiments were carried out after three passages.

### 2.5. Phytochemical Screening of *Piper capense*

The main classes of secondary metabolites, alkaloids (Mayer's tests), sterols (Salkowski's test), polyphenols (ferric chloride test), tannins (gelatin test), saponins (foam test), flavonoids (aluminum chloride test), triterpenes (Liebermann–Burchard's test), and anthraquinones (Borntrager's test), have been investigated following the phytochemical methods as described in [34, 35].

### 2.6. *In Vitro* Evaluation

#### 2.6.1. MTT Assay

The assay was carried out following the experimental protocol described in [36]. Briefly, 100 *µ*L of culture medium at a density of 1 × 10^4^ cells per well in a 96-well plate was exposed to 100 *µ*L increasing concentrations of PCFE (10–1000 *µ*g/mL) for 24 hours. 10 *µ*L of 5 mg/mL MTT (3-(4, 5-dimethylthiazol-2-yl)-2, 5diphenyltetrazolium bromide) was added to each well and incubated for 2 hours at 37°C. After incubation, 100 *µ*L DMSO was added to each well and the absorbance was measured at 570 nm using a microtitre plate reader (Tecan Infinite M200). Results were expressed as a percentage of viable cells as compared to 100% representing control cells. The IC_50_ value was calculated using nonlinear regression (curve fit) followed by log (inhibition) vs. response equation in GraphPad Prism software. The amount of drug was plotted on the *X*-axis as the log of drug concentration and OD was plotted on the *Y*-axis.

#### 2.6.2. Clonogenic Assay

B16-F10 cells were seeded at a very low number (500 cells in 2 mL/well) in six-well plates. After 24 h, they were treated with the optimum concentration of PCFE (100 *µ*g/mL) alone and in combination with dacarbazine at 1000 *µ*g/mL [37] and incubated for 72 hours. The colonies were fixed with methanol and stained with Harry's hematoxylin. The number of colonies defined as >50 cells/colony was counted under the bright field of a light microscope (Leica DM1000, Germany) at 200x magnification [38].

#### 2.6.3. Wound Healing Assay

The wound healing assay was performed in a 6-well plate following the experimental protocol described in [39]. 2 mL of B16-F10  cells was cultured in DMEM without serum to minimize cellular proliferation. Wounds were generated by scratching the 90% confluent cell monolayer with a sterile 200 *µ*l pipette tip. The unattached cells were washed away with PBS, and cells were treated with PCFE (100 *µ*g/mL) alone and in combination with dacarbazine (1000 *µ*g/mL) diluted in DMEM. Images of the wounds were acquired every 24 h under a phase-contrast microscope. The scratch area was measured using Wound Healing Tool in Image J software and relative scratch closure was calculated as the average length of the relative scratch gap based on the change in the scratch area at time zero.

#### 2.6.4. Immunofluorescence Staining (IFS)

For immunofluorescence analysis, 2 mL of B16-F10 cells in DMEM culture media was seeded on coverslips in six-well plates and cultured overnight. Cells were treated with PCFE (100 *µ*g/mL) alone and in combination with dacarbazine (1000 *µ*g/mL) for 24 h. The coverslips were fixed with methanol and incubated for 1 hour with 1 : 400 dilution of primary antibody against vimentin, E-cadherin, and CD133 followed by incubation with goat anti-mouse IgG-FITC (green) and goat anti-mouse IgG-PE (red) at 1 : 300 dilutions [40]. Coverslips were mounted with glycerol, and images were captured with a fluorescence microscope (Leica DM4000 B, Germany).

#### 2.6.5. Western Blot Analysis

Briefly, 2 mL of B16-F10 cells was seeded in six-well plates and treated with individual and combination doses of PCFE (100 *µ*g/mL) and dacarbazine for 24 hours. After incubation, treated cells were lysed in cold western blot lysis buffer (15 mM Tris, 2 mM EDTA, 50 mM 2-mercaptoethanol, 20% glycerol, 0.1% Triton X-100, 1 mM PMSF, 1 mM sodium fluoride, 1 mM sodium orthovanadate, 1 *µ*g/mL aprotinin, 1 *µ*g/mL leupeptin, and 1 *µ*g/mL pepstatin) for 5 minutes at room temperature. Cell lysates were sonicated and centrifuged at 13500 rpm for 15 minutes. Protein samples (50 *µ*g per lane) were thereafter resolved on a 10% sodium dodecyl sulfate-polyacrylamide gel (SDS-PAGE), blotted onto nitrocellulose membranes, blocked in TBS-T (0.1% Triton in 1xTBS), and probed with primary antibodies (E-cadherin, vimentin, CD133, and actin) overnight at 4°C. The membranes were incubated with the appropriate horseradish peroxidase-conjugated secondary antibodies. The immunoreactive protein bands were developed by an enhanced chemiluminescence kit (BioVision ECL Western Blot Substrate) and the immunoreactive bands were analyzed by using Image Lab software (Bio-Rad, GS 800) [41].

### 2.7. *In Vivo* Evaluation

#### 2.7.1. Experimental Animals

The male mice of strain C57BL/6J (aged 4-5 weeks; weight 22–25 g) were reared in the Animal House Department of the Chittaranjan National Cancer Institute in appropriate conditions with an alternating light-dark cycle of 12 hours and controlled temperature (22 ± 2°C). They were fed with a standard mouse diet and received water and feed ad libitum. The mice were acclimatized for seven days before being divided into different subgroups according to well-defined criteria before the onset of each experiment. The distribution of the mice was based on their body weight and tumor size as well. The animals were treated according to the recommendations of the Institutional Animal Ethics Committee (IAEC) of the Chittaranjan National Cancer Institute, Kolkata, just as the protocols used in this study were approved by this committee (Project no. IAEC-1774/NM-14/2019/9; 25.06.2019).

#### 2.7.2. Evaluation of the Effect of PCFE and Dacarbazine on Tumor Development in Mice

To check the effect of PCFE on solid melanoma tumor tissue, B16-F10 cells were administered in C57BL/6J mice. Five- to six-week-old C57BL/6J mice were subcutaneously injected with 2.5 × 10^4^ B16-F10 cells (in 200 *μ*L of PBS) into the right dorsolateral flanks. Treatment with the PCFE began once the tumors were visible and reached an average volume of 50 to 70 mm^3^. Animals were separated into six different groups (5 mice per group) receiving PCFE 50, 100, 150, 200, and 250 mg/kg body weight (b.w.), respectively, and control group. The choice of doses ≤250 mg/kg was made on the basis of the results of the acute and subchronic toxicity studies of PCFA carried out by Wamba and collaborators which highlighted in their work the nontoxic effects of PCFE at a dose of 250 mg/kg b.w./day and the toxic effects of PCFE at high doses [42]. After 15 days of intraperitoneal treatment, tumor size was determined and animals were sacrificed by cervical dislocation [43]. Tumors were collected for further analysis.

#### 2.7.3. Combination Treatment

In order to check the effect of dacarbazine alone and in combination with PCFE in solid melanoma tumor, the experiment was designed in 5 groups. The first group was treated with dacarbazine at 80 mg/kg body weight [37]; second, third, and fourth groups were treated with different doses of PCFE 100, 150, and 200 mg/kg b.w., respectively, along with dacarbazine, and the fifth group was considered as a control. The treatment was carried out over a period of seven days; on the 8th day, all animals were sacrificed. After determining the size of the tumor for each treatment group, the tumor tissues of the different groups were treated specifically for further experiments.

#### 2.7.4. CD31 Immunohistochemistry with PAS Staining for Identification of VM Tubes

Formalin-fixed paraffin-embedded (FFPE) sections (5 *µ*m) were kept in xylene for 20 minutes at 55°C and later dipped and deparaffinised in xylene for 10 min (3 changes). The tissue sections were rehydrated with various grades of alcohol. For CD31 expression, an immunochemistry study was performed according to the standard protocol as mentioned in the immunoperoxidase secondary detection system (DAB 150, Merck Millipore). After staining with DAB, the tissues were incubated with a freshly prepared periodic acid solution (5 mg/mL) for 15 min at room temperature [44]. The slides were treated with Schiff base for 20–30 min in dark and counterstained with Harris hematoxylin. Pink stained VM tubes in the sections were photographed using a bright-field microscope (Leica DM1000, Germany).

#### 2.7.5. Qualitative and Quantitative Estimation of the Markers that Play a Key Role in the VM

A qualitative analysis was made with the aid of immunofluorescence staining following the experimental protocol previously described [40]. The tissues were rehydrated with different grades of alcohol, blocked with a blocking buffer solution (1% bovine fetal serum (FBS), and incubated separately with primary antibodies anti-E-cadherin and anti-vimentin, followed by treatment with secondary antibodies marked PE and FITC. 4′, 6-Diamino-2-phenylindole (DAPI) (1 mg/mL; 1 : 10000) was used for nuclear staining. After mounting the slides, images were captured using a Leica BM 4000B fluorescence microscope (Germany). The alteration of EMT was evaluated by targeting E-cadherin, vimentin, and CD133 in PCFE alone and in combination with dacarbazine-treated tumor tissues. The mice melanoma tissues were homogenized and lysed (UP200S) in a western blot lysis buffer and centrifuged at 13500 rpm for 15 minutes (Thermo Scientific Biofuge Stratos). The lysate thus obtained was separated on SDS-PAGE and probed with desired primary and secondary antibodies.

#### 2.7.6. CD31 Immunohistochemistry with PAS Staining for Identification of Microvessel Density

To evaluate microvessel density for angiogenic activity in PCFE and dacarbazine-treated tumor tissues, CD31 immunohistochemistry with PAS staining was performed according to the standard protocol previously described [45]. The diameters of the microvessel were measured and their numbers were estimated after skeletonization of the endothelium in three different fields using ImageJ software.

### 2.8. Statistical Analysis

Statistical analyses were performed with GraphPad Prism 5 software. Representative data from three independent experiments are shown as mean value ± SEM. One-way analysis of variance (ANOVA) followed by post hoc Tukey's test was used to determine the significance of the difference between mean values relative to the control. The *p* value was calculated to determine significant differences (*p* value <0.05).

## 3. Results

### 3.1. Qualitative Phytochemical Composition of PCFE

The results of the phytochemical screening reported in Table 1 revealed the presence of alkaloids, polyphenols, saponins, tannins, and sterols in the plant extract while secondary metabolites such as anthraquinones, flavonoids, and triterpenes were absent in the plant extract.

### 3.2. PCFE Inhibits the Proliferation of B16-F10 Melanoma Cell Line

#### 3.2.1. MTT Assay

**Figure 1:**
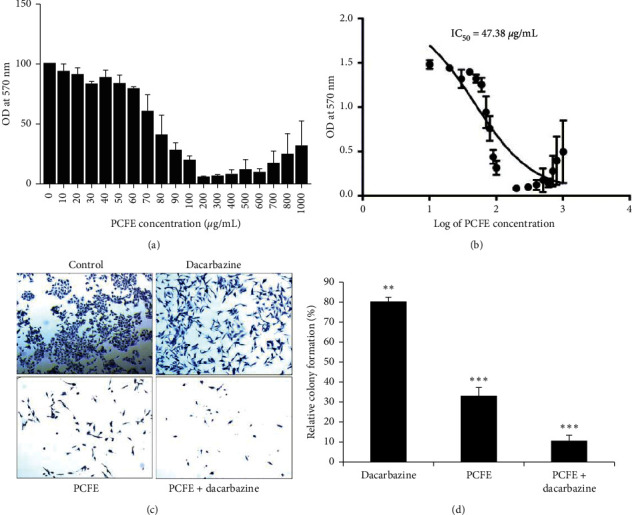
Cytotoxic effect of PCFE in B16-F10 cells. The percentage viability of PCFE-treated B16-F10 cells was calculated as OD of the drug-treated sample/OD of the nontreated sample × 100 (a). IC_50_ of melanoma cells treated with increasing concentration of PCFE was calculated as 47.38 *µ*g/ml (b). PCFE alone and in combination with dacarbazine significantly induced inhibition of colony formation and development of B16-F10 cells *in vitro* (c, d). Statistical significance of each treatment group with untreated control is analyzed by one-way ANOVA test (*p*_ANOVA _<_ _0.0001) followed by post hoc Tukey's test. Data are represented as mean ± SD of triplicate determinations from their independent experiments with ^*∗*^*p* value <0.05, ^*∗∗*^*p* value <0.01, and ^*∗∗∗*^*p* value <0.0001 versus untreated control and among each group.

To investigate the role of PCFE and dacarbazine on B16-F10 melanoma cells, MTT assay was performed. B16-F10 cells were treated with increasing concentrations of PCFE (10 to 1000 mg/mL) for 24 hours and subjected to MTT assay. The result showed a decrease in the viability of B16-F10 cells as compared to the control (Figure 1(a)). The IC_50_ value of treated B16-F10 cells was calculated to be 47.38 *µ*g/mL (Figure 1(b)).

#### 3.2.2. Colony Formation Assay

To further confirm the cytotoxicity of PCFE on melanoma cells, colony formation assay was performed. Cells were treated with single and combined doses of PCFE and dacarbazine for 24 hours. Result revealed that PCFE alone and in combination with dacarbazine significantly (*p* < 0.0001; *F* ratio = 244.3; *R*^2^ = 0.989) decreased the number of colonies in B16-F10 cells up to 35% and 12%, respectively (Figures 1(c) and 1(d)).

### 3.3. PCFE Inhibits EMT in B16-F10 Melanoma Cells

#### 3.3.1. Wound Healing Assay

**Figure 2:**
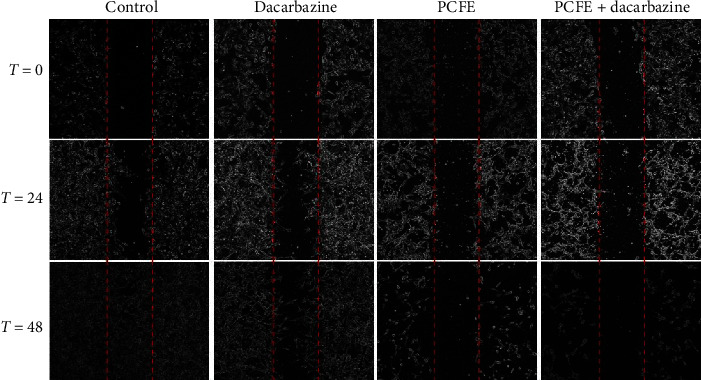
Antimigratory effect of PCFE on B16-F10. PCFE alone and in combination with dacarbazine prevents cell migration as compared to the control.

Wound healing assay showed that PCFE (100 *µ*g/mL) and dacarbazine (1000 *µ*g/mL) decreased the invasion and migration of the B16-F10 melanoma cells. However, PCFE treatment and combination treatment inhibit wound healing after 48 hours (Figure 2).

#### 3.3.2. Immunofluorescence Staining

**Figure 3:**
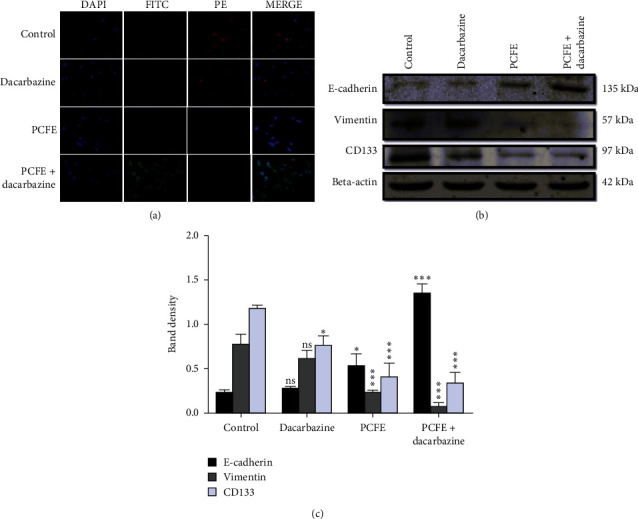
PCFE treatment modulates the expression of EMT markers *in vitro*. PCFE alone and in combination with dacarbazine upregulates the expression of FITC-tagged (green) E-cadherin and downregulates PE-tagged (red) vimentin of B16-F10 cells as compared to the untreated control (a). Western blot analysis and densitometric plot also showed significant upregulation of E-cadherin and downregulation of vimentin as well as CD133 (b, c). Statistical significance of each treatment group with untreated control is analyzed by one-way ANOVA test (*p*_ANOVA_ < 0.0001) followed by post hoc Tukey's test. Data are represented as mean ± SD of triplicate determinations from their independent experiments; ns: not significant, ^*∗*^*p* value <0.05, ^*∗∗*^*p* value <0.01, and ^*∗∗∗*^*p* value <0.0001 versus untreated control and among each group.

In order to evaluate the effect of PCFE and dacarbazine on EMT of B16-F10 cells, immunofluorescence assay was performed. Fluorescence intensity was quantified and represented as a percentage of intensity. The results revealed that PCFE at a concentration of 100 *µ*g/mL downregulates the expression of the vimentin and upregulates E-cadherin protein expression in B16-F10 cells. However, treatment with dacarbazine (1000 mg/mL) showed strong immunopositive expressions of vimentin and no detectable E-cadherin expression in B16-F10 cells. Conversely, in the combination treatment (PCFE 100 *µ*g/mL and dacarbazine 1000 *µ*g/mL), detectable expressions of E-cadherin and moderate immunopositive expression of vimentin were observed (Figure 3(a)).

#### 3.3.3. Western Blot Analysis

Western blot analysis was performed to evaluate the expression of E-cadherin, vimentin, and CD133. The results obtained showed a significant decrease in the expression of the vimentin (*p* < 0.0001; *F* ratio = 51.86; *R*^2^ = 0.9511) and CD133 (*p* < 0.0001; *F* ratio = 33.74; *R*^2^ = 0.9268) proteins compared to the control (Figure 3(b)). However, the expression of vimentin with dacarbazine treatment was not statistically significant with the control (calculated by Tukey's multiple comparison test) (Figure 3(c)). But we observed significant downregulation with PCFE alone at a concentration of 100 *µ*g/mL and in association with dacarbazine. Conversely, we observed a significant increase in the expression of E-cadherin protein (*p* < 0.0001; *F* ratio = 102.4; *R*^2^ = 0.9746) at different treatment concentrations compared to the progressive control as described progressively with effective upregulation at the treatment dose corresponding to the association between PCFE and dacarbazine. PCFE extract alone or in association with dacarbazine used in this work results in a downregulation of vimentin and CD133 proteins and an upregulation of the E-cadherin protein compared to the control (Figures 3(b) and 3(c)).

### 3.4. Antitumoral Activity of PCFE and Dacarbazine on B16-F10 Melanoma in Mice

#### 3.4.1. PCFE Reduces Tumor Size in B16-F10 Melanoma Mice

**Figure 4:**
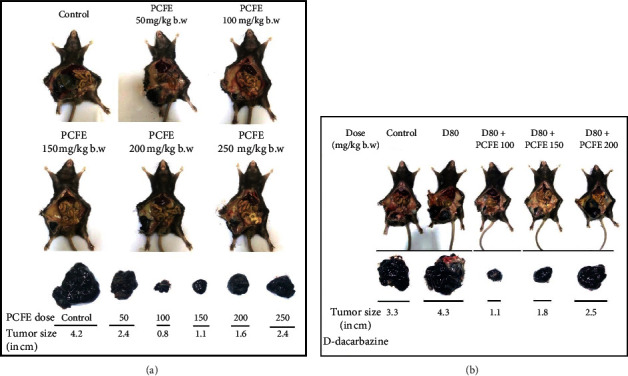
PCFE alone and in combination with dacarbazine causes a decrease in tumor size of B16-F10 melanoma mice. The treatment of melanoma mice with various doses of PCFE alone (a) and in combination with dacarbazine (b). PCFE alone at 100 mg/kg b.w. and in combination with dacarbazine showed a maximum decrease in tumor size as compared to the untreated control and other treatment regimes.

An increase in tumor size was observed in animals treated with dacarbazine (4.0 × 4.3 cm^2^) compared to control animals (3.3 × 3.1 cm^2^). In contrast, there was a remarkable decrease in tumor size in animals treated with different doses of PCFE: 50 mg/kg (2.4 × 2.6 cm^2^), 100 mg/kg (0.8 × 1.0 cm^2^), 150 mg/kg (1.0 × 1.1 cm^2^), 200 mg/kg (1.6 × 1.7 cm^2^), and 250 mg/kg (2.4 × 2.5 cm^2^). PCFE at different concentrations in combination with dacarbazine at 80 mg/kg showed remarkable inhibition in tumor size: dacarbazine + 100 mg/kg dose (1.1 × 1.3 cm^2^), dacarbazine + 150 mg/kg dose (1.9 × 1.8 cm^2^), and dacarbazine + 200 mg/kg dose (2.8 × 2.5 cm^2^). PCFE at the dose of 100 mg/kg b.w. showed maximum antitumor effect alone in melanoma mice as well as in combination with dacarbazine at 80 mg/kg b.w (Figures 4(a) and 4(b)).

#### 3.4.2. PCFE Inhibits the Formation and/or Development of Vasculogenic Mimicry

**Figure 5:**
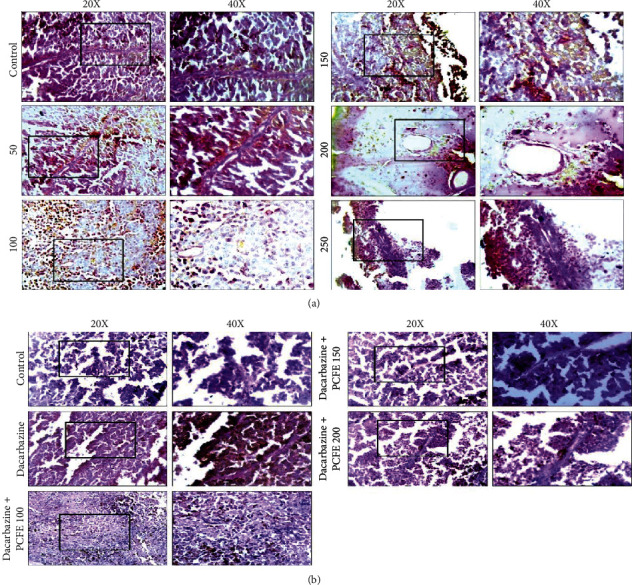
Effect of PCFE on the development of vasculogenic mimicry. CD31-PAS immunohistochemistry result showed PCFE alone (a) and in association with dacarbazine (b) inhibits the formation and development of vasculogenic mimicry in B16-F10 melanoma mice.

CD31-PAS immunohistochemistry results of dacarbazine-treated tumor tissue isolated from mice melanoma showed enormous VM tubes. However, different doses of PCFE alone and in combination with dacarbazine treatment showed inhibition of vasculogenic mimicry tubes (CD31 negative/PAS positive). 100 mg/kg b.w. doses of PCFE alone and in the presence of dacarbazine showed a significant decrease in the tumor size and therefore a decrease in the number of tubes at different doses of treatment (Figures 5(a) and 5(b)).

#### 3.4.3. Qualitative and Quantitative Effect of PCFE and Dacarbazine on the Expression of the Markers that Play a Key Role in the VM and EMT

**Figure 6:**
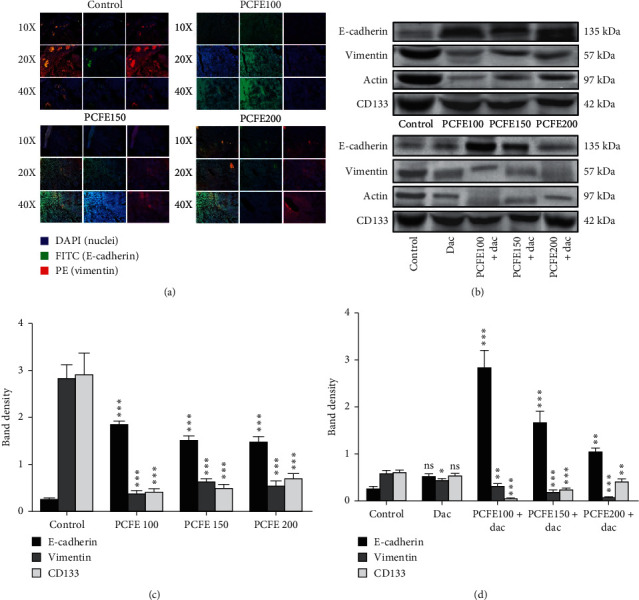
Qualitative and quantitative effect of PCFE on the expression of the markers that play a key role in the VM and EMT. Immunofluorescence study with various individual treatments of PCFE and in combination with dacarbazine in mice melanoma tumor sample showed an increase in the expression of E-cadherin and decrease in vimentin (a). Western blot analysis (b) and its densitometry plot (c, d) also showed significant upregulation of E-cadherin and downregulation of vimentin and CD133 in tumor tissue lysate of mice melanoma. Statistical significance of each treatment group with untreated control is analyzed by one-way ANOVA test (*p*_ANOVA_ < 0.0001) followed by post hoc Tukey's test. Data are represented as mean ± SD of triplicate determinations from their independent experiments with ^*∗*^*p* value <0.05, ^*∗∗*^*p* value <0.01, and ^*∗∗∗*^*p* value <0.0001versus untreated control and among each group.

The immunofluorescence carried out on the corresponding tissue sections, each at a precise dose, made it possible to demonstrate the effect of different doses of PCFE alone and in association with dacarbazine on protein expression. These proteins are involved in the heart of EMT, especially in the inhibition of the expression of vimentin (progressive reduction of the red color) and activation of the expression of E-cadherin (progressive increase in green color), dose dependently in groups of animals treated with PCFE compared to the control group (infected and untreated group). 100 mg/kg dose of PCFE was found to be the dose with the best ability to regulate the expression of these proteins both when administered individually and in combination with dacarbazine (Figure 6(a)). Similar results were obtained from western blot analysis of mice melanoma tumor lysates treated with different doses of PCFE which showed significant downregulation of CD133 (*p* < 0.0001; *F* ratio = 70.74; *R*^2^ = 0.963) and vimentin (*p* < 0.0001; *F* ratio = 152; *R*^2^ = 0.982) and upregulation of E-cadherin expression (*p* < 0.0001; *F* ratio = 158.1; *R*^2^ = 0.983) (Figures 6(b) and 6(c)). However, a better regulation was observed in terms of expression at the dose of 100 mg/kg individually and in combination with dacarbazine (Figure 6(d)).

#### 3.4.4. Effect of PCFE and Dacarbazine on Microvessel Density

**Figure 7:**
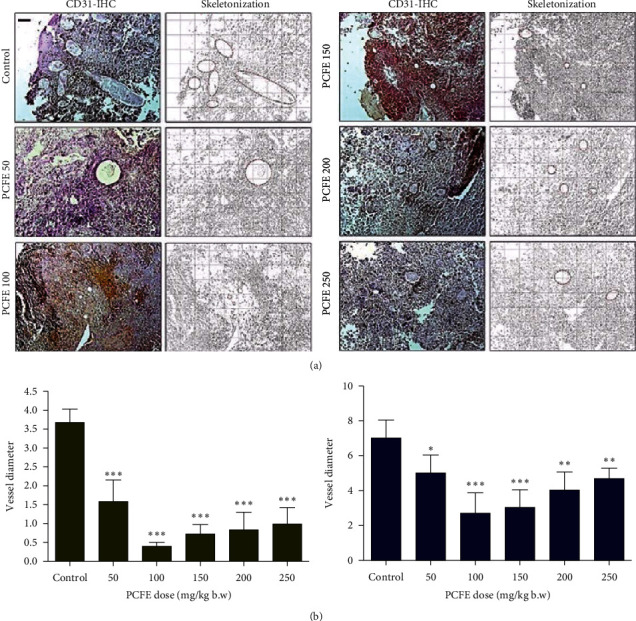
Effect of PCFE on microvessel density. PCFE alone at different doses effectively reduces microvessel size and density in B16-F10 melanoma mice.

**Figure 8:**
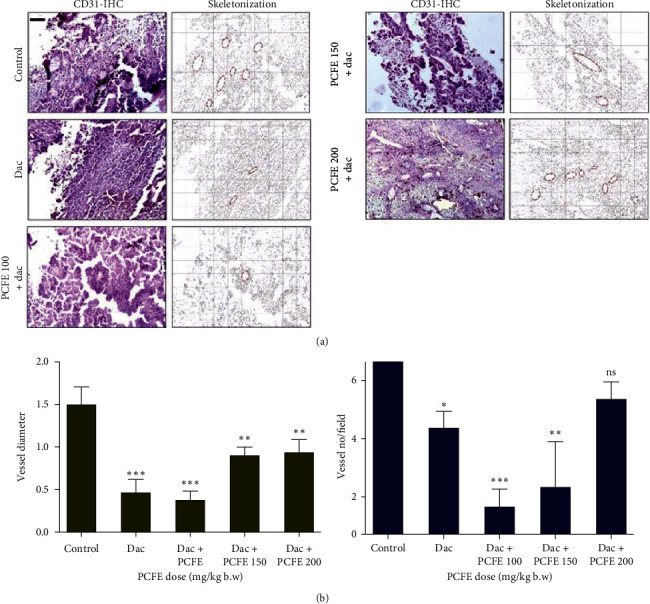
Effect of PCFE in association with dacarbazine on microvessel density. PCFE at different doses in combination with dacarbazine effectively reduces microvessel size and density in B16-F10 melanoma mice.

This test was performed in order to evaluate the effect of different doses of PCFE on the antigenic vessels present in the tumors collected from different groups of animals. The treatment of animals with the PCFE resulted in a progressive and significant decrease in the number and diameter of the different antiangiogenic vessels in different groups of animals tested and those treated more intensely treated with the 100 mg/kg dose (Figures 7 and 8).

## 4. Discussion

According to the International Agency for Research on Cancer (IARC) in 2018, the global cancer burden was estimated at 18.1 million for new cases against 9.6 million cancer deaths. Both metastatic and nonmetastatic melanomas have over time developed resistance to conventional anticancer drugs, which are nowadays classified as a public health hazard with reference to their relatively high mortality rate. There is therefore an urgent need to find new effective, low-toxicity substances, and given that phytochemicals are a poorly explored field makes it an unexplored gold mine for researchers. *Piper capense* is a food plant used in Cameroon and other countries in the world in the preparation of dishes and also for its therapeutic virtues [46, 47]. This plant species found in the natural flora of Cameroon has already been the subject of several *in vitro* studies on its anticancer activities on several cancer cell lines [28, 48]. Nevertheless, no reports in the literature have so far shown activities of this plant on melanoma both *in vitro* and *in vivo*, hence the essence of this work.

Several classes of secondary metabolites in plants are known to have cytotoxic and antitumor activities, including alkaloids, polyphenols, saponins, tannins, and sterols [49, 50]. The presence of alkaloids, polyphenols, saponins, tannins, and sterols in the methanol extract of *Piper capense* may justify its anticancer activities *in vitro* and *in vivo* observed in this work. The phytochemical screening results obtained in this study corroborate those of Fankam et al. [51].

Research on new drugs focuses on substances targeting a specific metabolic pathway or acting on a molecular target with a key role in the survival of cancer stem cells (CSCs), especially those with the ability to restore the expression of proteins involved in EMT. Epithelial-mesenchymal transition is a cellular process in which cells lose their epithelial characteristics and acquire mesenchymal characteristics. EMT has been associated with various tumor functions, including tumor initiation, malignant progression, tumor tenacity, tumor cell migration, metastasis, and treatment resistance [52, 53] and is often defined by downregulation of the epithelial marker E-cadherin and upregulation of the mesenchymal markers vimentin and CD133 [54].

The results obtained in this work show the ability of PCFE to induce a cytotoxic effect on B16-F10 cells and the ability of PCFE alone and in combination with dacarbazine to prevent and/or inhibit colony formation and also for the inhibition of wound healing of B16-F10 melanoma cells and to the reversal of markers involved in EMT by modification of their expression *in vitro*. All these activities could be attributed to the presence of various secondary metabolites with proven anticancer activity in this plant extract [55]. Many works have demonstrated the antiproliferative effect of secondary metabolites such as alkaloids, phenolic compounds, and triterpenes [28, 56, 57]. PCFE alone and in combination with dacarbazine-induced shrinkage of tumor size in animals by inhibiting the development of VM tubes and microvessel density. It also restored the expression of proteins involved in EMT (downregulation of CD133 and vimentin markers and upregulation of E-cadherin) both *in vitro* and *in vivo*. These activities could be due to the presence of the secondary metabolites endowed with anticancer activity in the extract. These results corroborate those of other previous works that have shown that spices possess cytotoxic activities either by induction of apoptosis or by cell cycle arrest at a specific phase [28, 58] and action on EMT markers *in vitro* and *in vivo* [59–61].

The work done by Woguen et al. [48] on the evaluation of the phytochemical composition of essential oil of fruits of *P. capense* by GC-MS revealed the presence of two major compounds (*β-*pinene and (E)-caryophyllene) with good cytotoxic activities. *β-*Pinene and *(E)-*caryophyllene are compounds belonging to the class of phenolics and terpenoids, respectively; the latter were identified in PCFE. Phenolics and terpenoids are classes of secondary metabolites with antiproliferative and antitumor activities [48, 56]. The presence of these classes of metabolites in PCFE may account for the *in vitro* and *in vivo* anticancer activities observed in this study.

Some compounds like piperine found in alkaloids having anticancer activities *in vitro* and *in vivo* as documented in the literature are also found in plants belonging to the genus *Piper* and the species *Piper nigrum*, *Piper longum*, and *Piper capense* [61–63]. In view of the above, the similarities between the results with the methanol extract of *Piper capense* obtained in this study and those with piperine obtained in other studies lead us to suggest that alkaloids (one group of secondary metabolite highlighted in PCFE) could also be responsible for the activities observed in this study. The results of this study showed the capacity of PCFE to inhibit the formation and development of VM tubes which are indeed neoformed vessels which play a role in supplying the cancerous cells with nutrients at the level of the primary tumor [64]. The inhibition of the development of these VM tubes by PCFE accompanied by the restoration of the expression of proteins involved in EMT could justify the decrease in the size of the tumor because it lacks a supply route. Also, the antiangiogenic activities of this plant extract were observed in this study as shown previously [65]. These aforementioned activities demonstrate and sufficiently justify the antiproliferative activity of PCFE and could be due to the possible presence of alkaloids in this plant.

The work of Greenshields et al. [66] demonstrates the ability of piperine to inhibit the growth of triple breast cancer xenografts in immune-deficient mice corroborating the results of this study which was carried out on moles of mice of C57BL/6J strains with melanomas. Makhov et al. [67] showed a remarkable effect of the association of an anticancer agent (docetaxel) with piperine through the improvement of the antitumor activity in the xenograft model of human castration-resistant prostate cancer. This is consistent with the results obtained in our study given that a remarkable improvement in the activity of dacarbazine (conventional chemotherapeutic drug of melanoma) administered in association with the PCFE was noted both *in vitro* and *in vivo.* A justification for this activity could be due to the possible presence of one or many active compounds that can be found in the different groups of secondary metabolite that we have highlighted in PCFA.

## 5. Conclusion

In conclusion, this study, which is the first of its kind to evaluate the anticancer activity of the methanol extract of *Piper capense* fruit against melanoma *in vitro* and *in vivo*, clearly demonstrates the ability of PCFE to inhibit cell proliferation alone and in combination with dacarbazine *in vitro*, to induce the shrinkage of the melanoma tumor size in mice by modification of expression of the markers involved in EMT through downregulation of CD133 and vimentin markers and up-regulation of the E-cadherin marker *in vivo* in melanoma models with the best effect at 100 mg/kg b.w. These results coupled with those in the literature indicate that the *Piper capense* plant is a very good candidate plant for the formulation of phytomedicines in the treatment of melanoma. One of the limitations of this study is the characterization of the phytochemical constituents and mostly the bioactive as well as potentially toxic constituents of the tested methanol extract. However, this work is ongoing and constitutes the aim of our further investigations.

## Figures and Tables

**Table 1:** Phytochemical composition of PCFE.

Chemical classes	*Piper capense* extract
Extractive yield (%)	12.80

Alkaloids	+
Anthraquinones	−
Flavonoids	−
Polyphenols	+
Saponins	+
Tannins	+
Sterols	+
Triterpenes	−

(+): present; (−): absent; yield is obtained by the ratio of the mass of the extract to the methanol obtained to the mass of the plant powder.

## Data Availability

All data generated or analyzed during this study are included in this published article.

## Abbreviations

PCFE: *Piper capense* fruit extract
MTT: 3-(4, 5-Dimethylthiazol-2-yl)-2, 5-diphenyl tetrazolium bromide
EMT: Epithelial-to-mesenchymal transition
DAB: 3′3-Diaminobenzidine
DMEM: Dulbecco's modified Eagle medium
FBS: Fetal bovine serum
VM: Vasculogenic mimicry
MIC: Minimal inhibitory concentration
ABC: ATP-binding cassette
CSC: Cancer stem cell
IC_50_: Inhibitory concentration 50%
WHO: World Health Organization.
